# The Effect of Exercise on Cardiometabolic Risk Factors in Women with Polycystic Ovary Syndrome: A Systematic Review and Meta-Analysis

**DOI:** 10.3390/ijerph19031386

**Published:** 2022-01-26

**Authors:** Annabelle Breyley-Smith, Aya Mousa, Helena J. Teede, Nathan A. Johnson, Angelo Sabag

**Affiliations:** 1 Faculty of Medicine and Health, Discipline of Exercise and Sports Science, University of Sydney, Camperdown, NSW 2006, Australia; annabelleamber8@gmail.com (A.B.-S.); nathan.johnson@sydney.edu.au (N.A.J.); 2 Monash Centre for Health Research and Implementation (MCHRI), School of Public Health and Preventive Medicine, Monash University, Clayton, VIC 3168, Australia; helena.teede@monash.edu; 3 NICM Health Research Institute, Western Sydney University, Westmead, NSW 2145, Australia

**Keywords:** women’s health, cardiometabolic health, cardiorespiratory fitness, waist circumference, physical activity

## Abstract

Background: Polycystic Ovary Syndrome (PCOS), a common endocrine disorder in women of reproductive age, increases the risk for cardiometabolic morbidity. While regular exercise is effective in reducing cardiometabolic risk, women with PCOS may experience condition-specific barriers to exercise thereby limiting its efficacy. Aim: To determine the effect of exercise on cardiometabolic risk factors in women with PCOS. Methods: Five databases (Cochrane, EMBASE, Medline, Scopus and SPORTDiscus) were searched up to December of 2021. Eligible studies included: a randomised controlled design; participants with a diagnosis of PCOS; aerobic and/or resistance exercise intervention lasting ≥4 weeks; cardiometabolic outcomes. Meta-analyses were performed to determine the effect of exercise versus non-exercising control on cardiometabolic outcomes. Results: Of the 4517 studies screened, 18 studies were analysed involving 593 participants. When compared with control, exercise significantly improved cardiorespiratory fitness (weighted mean difference {WMD} = 4.00 mL/kg/min, 95% CI: 2.61 to 5.40, *p* < 0.001) and waist circumference (WMD = −1.48 cm, 95% CI: −2.35 to −0.62, *p* = 0.001). Systolic blood pressure, fasting blood glucose, insulin resistance, and lipid profiles remained unchanged. Conclusions: Regular exercise may improve cardiorespiratory fitness and waist circumference in women with PCOS. Further large-scale studies are required to determine whether exercise interventions improve various biochemical and anthropometric parameters in women with PCOS and more severe cardiometabolic abnormalities.

## 1. Introduction

Polycystic Ovary Syndrome (PCOS) is a common endocrine disorder which is estimated to affect approximately one in every five women of reproductive age worldwide [1], with variation in prevalence depending on the population and diagnostic criteria used. The Rotterdam 2003 criteria, which is the most widely accepted diagnostic criteria, requires two of the following for a diagnosis of PCOS: oligo/anovulation, clinical and/or biochemical signs of hyperandrogenism, and polycystic ovaries. Furthermore, these characteristics are often associated with a range of symptoms such as hirsutism, infertility, acne, and overweight and obesity.

Women with PCOS are at an increased risk of developing the metabolic syndrome and its individual components, particularly increased waist circumference and elevated fasting glucose [2]. It is estimated that 35% of women with PCOS also present with overweight or obesity, with higher body mass index (BMI) related to an increased severity of symptoms such as hirsutism [3]. In addition, women with PCOS are at an increased risk of insulin resistance, which affects approximately 64% of this population [4]. Although not included in any diagnostic criteria, due to variability in assessment methodologies, insulin resistance underpins the aetiology of PCOS [2,5] and may contribute to the population’s higher risk of developing type 2 diabetes [6] and cardiovascular disease [7]. Furthermore, women with PCOS also present with impaired cardiorespiratory fitness [8], which may further exacerbate cardiometabolic risk. While there is currently no curative treatment for PCOS, management of the condition focuses on the improvement of symptoms, fertility (if desired), and cardiovascular and cardiometabolic risk.

In managing PCOS, lifestyle intervention involving dietary and exercise modification is recommended to improve quality of life as well as to ameliorate cardiovascular and metabolic perturbations that can arise as a result of the condition, with a strong focus on weight management [9]. Interestingly, exercise often elicits cardiometabolic benefits in the absence of significant weight loss [10], and as a result, may hold unique benefits for the management of cardiometabolic health in PCOS. Exercise prescription recommendations in women with PCOS are similar to those for the general population, as featured in the international evidence-based guidelines for PCOS [9], however, some of the evidence used to inform the exercise components is of low quality and comprised of non-randomised controlled trials. Furthermore, recent evidence has suggested that variations of aerobic exercise approaches, such as high-intensity interval training (HIIT), often lead to similar and, at times, greater improvements in cardiometabolic risk factors to higher volume moderate-intensity continuous training (MICT) despite often requiring less time commitment and expending less energy [10]. Consequently, the efficacy of such interventions requires further investigation in the context of PCOS. Finally, the increasing rate at which clinical trials regarding the effect of exercise on PCOS are published necessitates regular reviews and analyses of the literature to ensure that clinical practice remains relevant and informed. Therefore, the aim of this systematic review and meta-analysis is to utilise pooled data from randomised controlled trials (RCTs) to determine the effect of exercise on key cardiometabolic risk factors including cardiorespiratory fitness, waist circumference, systolic blood pressure, homeostatic model assessment of insulin resistance (HOMA-IR), fasting blood glucose, triglycerides, and high-density lipoprotein cholesterol (HDL-C) in women with PCOS. A further aim is to determine the independent effects of HIIT and MICT on relevant cardiometabolic outcomes when compared to control. 

## 2. Methods

This systematic review was conducted and reported based on the Preferred Reporting Items for Systematic Reviews and Meta-analysis statement (PRISMA) [11], and was prospectively registered on PROSPERO (CRD42020199438). 

### 2.1. Literature Search Strategy

A comprehensive electronic database search was conducted in MEDLINE (via Ovid), EMBASE, Cochrane Library, Scopus, and SPORTDiscus from inception to December 2021 using keywords related to PCOS and exercise. A search strategy was developed for MEDLINE and adapted for the subsequent databases searched. The strategy was performed using the following keywords and truncations, and was adapted, where necessary, to relevant databases: Polycystic Ovary Syndrome (Polycystic Ovary Syndrome* OR PCOS OR PCOD OR Polycystic Ovar* OR stein leventh* OR PCO) AND exercise (exercise* OR physical activit* OR physical fitness* OR walk* OR resistance train* OR muscle train* OR strength train* OR endurance train* OR interval train* OR intermittent train* OR swim* OR bicycl* OR cycling). 

Studies were limited, where possible, to those reported in English with human female subjects. The reference lists of eligible articles were searched for the identification of additional studies. Book sections, theses, film broadcasts, opinion articles or commentaries, observational studies, abstracts without adequate data, and reviews were excluded. 

### 2.2. Inclusion and Exclusion Criteria

#### 2.2.1. Participants

Eligible studies were those which included women with an average age of 18 years or older, of any activity level, with a diagnosis of PCOS based on Rotterdam 2003 diagnostic criteria [12], National Institute of Health 1990 diagnostic criteria [13] or Androgen Excess and PCOS Society 2006 criteria [14]. Trials were also included if diagnosis was self-reported or certified by a physician. Studies of participants with conditions of similar symptomatology to PCOS were excluded, including congenital adrenal hyperplasia, Cushing’s syndrome, hyperprolactinaemia, thyroid disease, and androgen secreting tumours. 

#### 2.2.2. Intervention

Eligible studies employed an RCT design which included a regular exercise training intervention (≥4 weeks) with modes of aerobic or resistance training, or a combination of the two. Interventions consisting of yoga, pilates or sporting activities were excluded.

#### 2.2.3. Comparator

Eligible studies employed a non-exercise or sham-exercise (e.g., stretching) group as a control. Studies with a concurrent treatment were included, provided that it was consistent across all groups in order to isolate the independent effect of exercise (e.g., exercise plus diet versus diet only). 

#### 2.2.4. Outcome

Eligible studies reported changes in one or more of the following outcomes: cardiorespiratory fitness measured as maximal or peak oxygen uptake (mL/kg/min), waist circumference (cm), systolic blood pressure (mmHg), HOMA-IR, fasting blood glucose (mmol/L or mg/dL), triglycerides (mmol/L or mg/dL) or HDL-C (mmol/L or mg/dL). Studies were included if they reported pre- and post-intervention mean ± standard deviation (SD) or change score. Results reported as standard error of the mean (SEM) were converted to SD using SD = SEM × square root of sample. Biochemical parameters reported in mg/dL were converted to mmol/L using the applicable formulas for each outcome.

### 2.3. Data Synthesis

After removing duplicates, two researchers (A.B. and A.S.) independently screened the search results against the eligibility criteria, and records which could not be eliminated by title or abstract were retrieved and reviewed in full. Disagreements regarding eligibility were resolved by a third researcher (N.A.J.). Attempts were made to contact the authors for additional information where needed. In instances where the authors were unresponsive, the studies were eliminated. Data regarding participant characteristics (age, BMI, and PCOS diagnostic criteria), exercise interventions (mode, frequency, intensity, session duration, intervention duration), additional interventions (dietary and/or pharmacological prescriptions), and pre- and post-intervention measures of cardiorespiratory fitness, waist circumference, systolic blood pressure, HOMA-IR, fasting blood glucose, triglycerides, and/or HDL-C were extracted. Aerobic exercise interventions could be further subcategorised into either MICT or HIIT according to classifications and guidelines published elsewhere [10,15]. All data were independently extracted by two researchers (A.B. and A.S.). Discrepancies were resolved via synchronous review of data presented in the original publications.

### 2.4. Data Analysis

The data were pooled using Comprehensive Meta-Analysis, Version 3 (Biostat Inc., Englewood, NJ, USA). The primary analyses involved determining the effect of exercise per se on cardiometabolic health outcomes. Random effects models were used and the weighted mean differences with 95% confidence intervals (CIs) were assessed. The variability of studies was determined using the *I*^2^ measure of consistency, which provides a measure of the amount of variability due to heterogeneity rather than sampling error. *I*^2^ values of ≤25, 26–74, and ≥75 were regarded as low, moderate, and high, respectively [16]. Assessment of publication bias was performed via examination of funnel plot asymmetry (precision vs. effect size) and using the Egger’s test. Sensitivity analyses were completed where studies revealed publication bias, or where the pooled results were significantly affected by the weighting of individual studies. Where there were three or more studies, sub-analyses were completed to assess the effect of HIIT vs. control and MICT vs. control on cardiometabolic outcomes.

### 2.5. Methodological Quality

Methodological quality and risk of bias of the included studies were assessed by two reviewers (A.B. and A.S.) using a modified Downs and Black Checklist [17], consisting of questions that addressed the ability of each study to clearly state its aims, participants, outcome measurements and interventions, accurately represent its participant groups, perform correct statistical analysis, and report their findings accurately. The scale was modified to include two additional criteria regarding exercise supervision and adherence to reflect their possible impact on the primary outcomes of the study (see Appendix A). A total of 29 questions were scored as no = 0, unable to determine = 0, or yes = 1, hence, the highest possible score for a study was 29. Discrepancies between reviewers were resolved via synchronous review of data presented in the original publications. Studies were classified as being of either low-, moderate-, or high-quality with respective scores of 0–10, 11–20, and 21–29. 

### 2.6. Risk of Bias Assessment and Certainty of Evidence

Studies were assessed for bias by one reviewer (A.S.) using the Cochrane Risk of Bias 2 tool, which is structured into a fixed set of domains of bias, including selection bias, performance bias, detection bias, attrition bias, reporting bias, and other bias [18]. Other bias was judged by assessing whether studies reported exercise adherence. 

The certainty of the evidence was assessed using the Grading of Recommendations, Assessment, Development, and Evaluation (GRADE) framework [19] by one reviewer (A.S.).

## 3. Results

**Figure 1:**
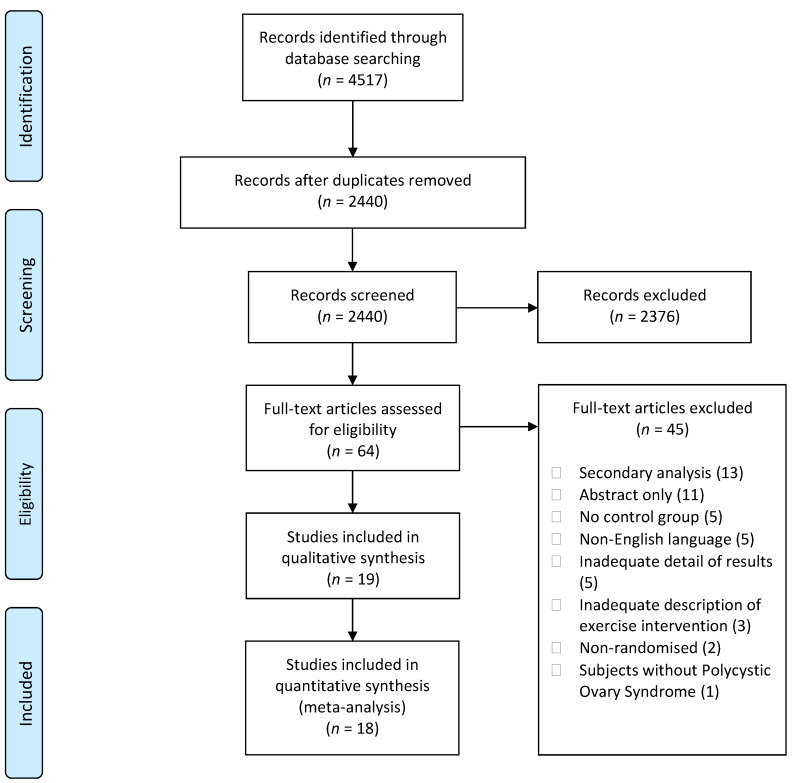
Preferred Reporting Items for Systematic Reviews and Meta-analyses (PRISMA) flow diagram.

A total of 4517 articles were identified in the database searches. Following the removal of duplicates and elimination of studies deemed ineligible, 19 studies were included in the systematic review of which 18 were pooled for meta-analysis (Figure 1). 

### 3.1. Participant Characteristics

Baseline characteristics for a total of 613 participants from the included studies are summarised in Table 1. Two studies reported different outcomes from the same clinical trial [20,21]. As a result, the number of participants from only one of these studies was used to tally the total number of participants included in this review. Mean age ranged from 24 to 32 years and mean BMI ranged from 21.8 to 41.3 kg/m^2^. All but two studies recruited individuals with overweight or obesity [22,23], and all but three studies classified PCOS using the Rotterdam 2003 diagnostic criteria [20,22,24]. Nine studies recruited inactive individuals [22,23,25,26,27,28,29,30,31], two recruited individuals with insulin resistance [31,32], and one recruited healthy weight individuals [23].

### 3.2. Intervention Characteristics

Exercise intervention characteristics are summarised in Table 2. Ten studies employed a MICT protocol [20,21,22,26,28,29,30,31,36,38], six studies employed a HIIT protocol [25,26,29,32,33,35], two studies employed a resistance training (RT) protocol [24,25], and three studies employed a concurrent MICT and RT protocol [23,27,30]. All studies included a non-exercising control for comparison, and most included a concurrent treatment consistent across all groups. Concurrent treatments included advice to maintain usual diets (*n* = 6) [22,25,28,29,32,38]; prescription of a high-protein, low-carbohydrate diet (*n* = 4) [23,30,31,37]; education regarding the importance of a healthy diet and/or physical activity (*n* = 4) [20,33,35,36]; 1 h weekly seminars/counselling regarding long-term nutritional and physical activity strategies (*n* = 2) [27,35]; a 600 kcal deficit per day [34], or prescription of 500 mg metformin taken three times per day (*n* = 1) [32]. The control group of one study was advised to adhere to at least 150 min of moderate-intensity exercise per week, without any follow-up during the intervention period [25]. Intervention durations ranged from 8 to 20 weeks, with the most common being 12 weeks (*n* = 6) [22,24,27,31,32,37]. Exercise frequency ranged from two to five days per week.

For MICT interventions, both alone and in combination with RT, the most common mode of exercise was walking and/or jogging outdoors or on a treadmill (*n* = 7) [20,22,27,28,29,30,36], followed by stationary cycling (*n* = 8) [20,21,22,27,31,36,37,38]. Some studies allowed participants to select their preferred aerobic exercise modality, which included either walking, running, cycling, or elliptical. For HIIT interventions, participants exercised on a treadmill [29]; on a cycle ergometer and/or running [35]; by performing aquatic exercises [32], and three studies allowed participants to select treadmill or outdoor walking/running and/or cycling [25,26,33]. All RT interventions, both alone or in combination with MICT, prescribed dynamic exercises performed with machine or free weights (*n* = 2) [24,30], resistance bands (*n* = 1) [23], or the participants’ own bodyweight (*n* = 1) [24] to target all muscle groups of the body. One study did not specify their prescribed exercises beyond “dynamic strength drills” [25].

For HIIT and MICT interventions, both alone or in combination with RT, aerobic exercise intensity was prescribed as a percentage of the participants’ maximal heart rate (HR_max_), or maximal or peak oxygen uptake (VO_2max_ or VO_2peak_, respectively). HIIT interventions involved high-intensity bouts lasting 20 to 240 s at intensities of 70 to 100% HR_max_, separated by resting periods of 10 to 180 s. MICT interventions involved intensities of 60 to 85% HR_max_, 50 to 65% VO_2peak_, 60 to 70% VO_2max_, and a heart rate of 120 beats per minute or greater. The intensities of RT interventions, while not consistently reported, were prescribed using a percentage of participants’ 1RM (one-repetition maximum) or RPE (rating of perceived exertion) scale from 1 to 10. RT interventions involved intensities of 50 to 75% 1RM, an RPE of 5 to 6 out of 10, or aimed to reach muscular fatigue at the end of each set.

The duration of HIIT and MICT sessions ranged from 30 to 50 min and 25 to 65 min for HIIT and MICT, respectively, including warm-up and cool-down if prescribed. RT exercises were performed for one to three sets of 8 to 15 repetitions each. Exercise was fully supervised in nine studies [22,23,27,28,29,31,33,37,38], weekly support through telephone calls was provided in three studies [20,21,36], and three included both supervised and unsupervised sessions [24,25,33]. The three remaining studies did not report supervision status [26,30,32]. 

Cardiorespiratory fitness was measured via analysis of expired respiratory gases (*n* = 5) [22,25,26,35] or indirectly (inferred—without gas analysis) (*n* = 8) [20,21,27,28,32,33,36,37] through either graded exercise tests, performed on a treadmill (*n* = 4) [22,25,28,33] or bicycle ergometer (*n* = 7) [20,21,27,35,36,37,38], or the 20-m Shuttle Run Test (*n* = 1) [32]. The results of these tests were reported as either VO_2max_ or VO_2peak_.

### 3.3. Methodological Quality, Risk of Bias, and Certainty of Evidence

The results of the methodological quality and risk of bias assessment are presented in Table 3. The scores ranged from 16 to 25, with an average of 20.8 ± 2.5. Six studies were classified as being of moderate quality [20,22,27,31,32,34], while the remainder were considered to be of high-quality.

All studies reported their main findings and variability estimates. Due to the nature of exercise trials, subjects were not blinded to their intervention group. The majority of studies reported their aims, outcomes, participant characteristics, interventions, principal confounders, *p* values, and accuracy of measures. Nine studies reported on adherence to exercise sessions, with an average adherence rate of 82% [22,23,24,25,26,28,29,35,37]. A further ten provided supervision for the exercising participants [23,24,25,27,28,29,31,33,37,38]. Seven of the 15 studies reported on adverse events, though none were recorded [20,24,25,26,29,31,37]. Four studies reported attempts to blind those measuring the main outcomes of the intervention [21,26,36,37]. 

**Figure 2:**
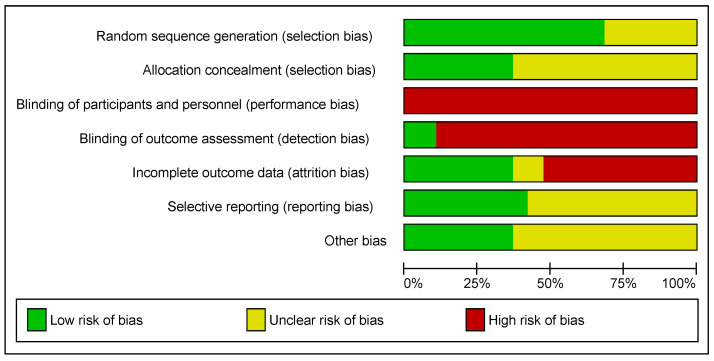
Risk of bias summary.

The results of the risk of bias assessment are summarised in Figure 2. Twelve studies scored an unclear or high risk of bias on five or more domains [20,21,24,27,28,30,31,32,33,35,36,38]. Two studies scored an unclear or high risk of bias on four domains [22,37]. Four studies scored an unclear or high risk of bias on three or less domains [23,25,26,29].

The level of certainty of the results produced are detailed in Table 4. There was a low certainty of evidence showing that exercise may result in higher cardiorespiratory fitness when compared to control. There was a very low certainty of evidence showing that exercise may reduce waist circumference when compared to control. There was a very low certainty of evidence showing that exercise is unlikely to induce any meaningful improvement in systolic blood pressure in normotensive women with PCOS when compared to control. There was a low level of certainty showing that exercise is unlikely to induce any meaningful improvement in HOMA-IR when compared to control. There was a low level of certainty that exercise is unlikely to result in any meaningful improvement in fasting blood glucose, blood triglycerides, or HDL-C in women with PCOS (and normal scores for these parameters) when compared to control. 

### 3.4. Meta-Analysis

Eighteen studies were included in meta-analysis, consisting of 593 participants. Of those eligible for meta-analysis, nine studies reported cardiorespiratory fitness in mL/kg/min [20,25,26,27,28,32,33,37,38]; twelve studies reported waist circumference in cm [21,23,24,25,26,27,28,29,30,33,35,37]; six studies reported systolic blood pressure in mmHg [21,23,26,28,30,37]; ten studies reported HOMA-IR [23,24,25,26,29,30,31,32,34,36]; eleven studies reported fasting blood glucose in mmol/L or mg/dL [23,24,25,26,28,29,30,31,34,36,37]; eight studies reported triglycerides in mmol/L or mg/dL [23,25,26,28,29,30,36,37], and eight studies reported HDL-C in mmol/L or mg/dL [23,25,26,28,29,30,36,37]. One study reported relevant outcomes as pre-intervention mean ± SD and relative change, and was consequently excluded from the meta-analysis [22].

#### 3.4.1. Cardiorespiratory Fitness

**Figure 3:**
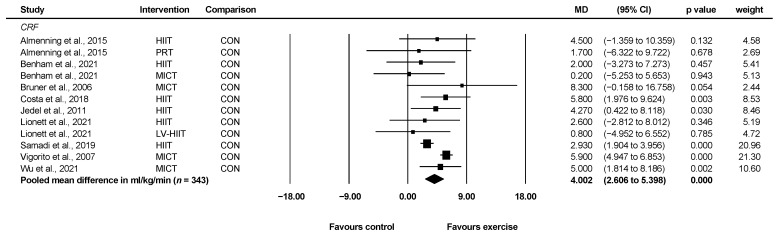
Effect of exercise vs. control for change in cardiorespiratory fitness. CI: confidence interval, CRF: cardiorespiratory fitness, HIIT: high-intensity interval training, LV-HIIT: low-volume high-intensity interval training, MD: mean difference, MICT: moderate-intensity continuous training, *PRT*: progressive resistance training.

Nine studies involving 343 unique participants reported cardiorespiratory fitness as VO_2max_ or VO_2peak_ in ml/kg/min [20,25,26,27,28,32,33,37,38]. Two studies reported cardiorespiratory fitness in mL/min and were consequently excluded from the pooled analysis [23,35]. When compared with control, exercise significantly improved cardiorespiratory fitness (WMD = 4.00 mL/kg/min, 95% CI: 2.61 to 5.40, *p* < 0.001, *I*^2^ = 54.77 (Figure 3). Two studies indicated potential publication bias following Egger’s test [32,37]. When these two studies were removed in sensitivity analyses, the results remained significant (WMD = 3.85 mL/kg/min, 95%CI: 2.32 to 5.38, *p* < 0.001, *I*^2^ = 0).

#### 3.4.2. Waist Circumference

**Figure 4:**
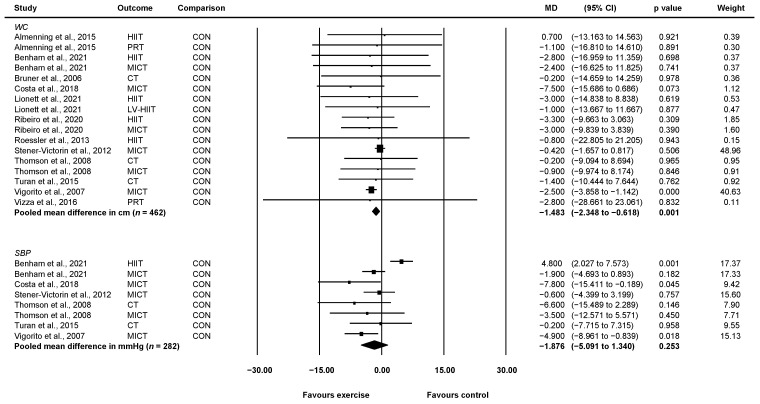
Effect of exercise vs. control for change in waist circumference and systolic blood pressure. CI: confidence interval, CT: concurrent training, HIIT: high-intensity interval training, LV-HIIT: low-volume high-intensity interval training, MD: mean difference, MICT: moderate-intensity continuous training, PRT: progressive resistance training, SBP: systolic blood pressure, WC: waist circumference.

Twelve studies involving 462 unique participants reported waist circumference in cm [21,23,24,25,26,27,28,29,30,33,35,37]. Compared with control, exercise significantly improved waist circumference (WMD = −1.48 cm, 95% CI: −2.35 to −0.62, *p* = 0.001, *I*^2^ = 0) (Figure 4). After removing 2 of 14 studies which weighed 48.96% and 40.63% [21,37], respectively, waist circumference decreased further but the results were no longer significant (WMD = −2.54 cm, 95%CI: −5.24 to 0.16, *p* = 0.066, *I*^2^ = 0).

#### 3.4.3. Systolic Blood Pressure

Six studies involving 282 unique participants reported systolic blood pressure in mmHg [21,23,26,28,30,37]. When compared with control, exercise did not significantly improve systolic blood pressure (WMD = −1.88 mmHg, 95% CI: −5.09 to 1.34, *p* = 0.253, *I*^2^ = 72.31) (Figure 4). Two groups from two separate studies indicated potential publication bias following Egger’s test [26,37]. After removing these, exercise significantly reduced systolic blood pressure when compared to control (WMD = −2.14 mmHg, 95% CI: −4.11 to −0.16, *p* = 0.034, *I*^2^ = 0).

#### 3.4.4. HOMA-IR

**Figure 5:**
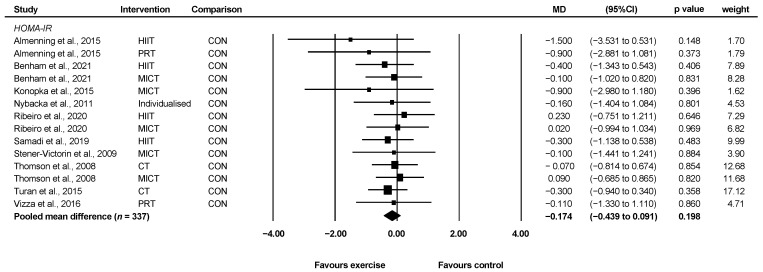
Effect of exercise vs. control for change in HOMA-IR. CI: confidence interval, CT: concurrent training, HIIT: high-intensity interval training, MICT: moderate-intensity continuous training, MD: mean difference, PRT: progressive resistance training.

Ten studies involving 337 unique participants reported HOMA-IR [23,24,25,26,29,30,31,32,34,36]. Exercise did not significantly improve HOMA-IR compared with control (WMD = −0.17, 95% CI: −0.44 to 0.09, *p* = 0.198, *I*^2^ = 0) (Figure 5). There was no publication bias detected following Egger’s test.

#### 3.4.5. Fasting Blood Glucose

**Figure 6:**
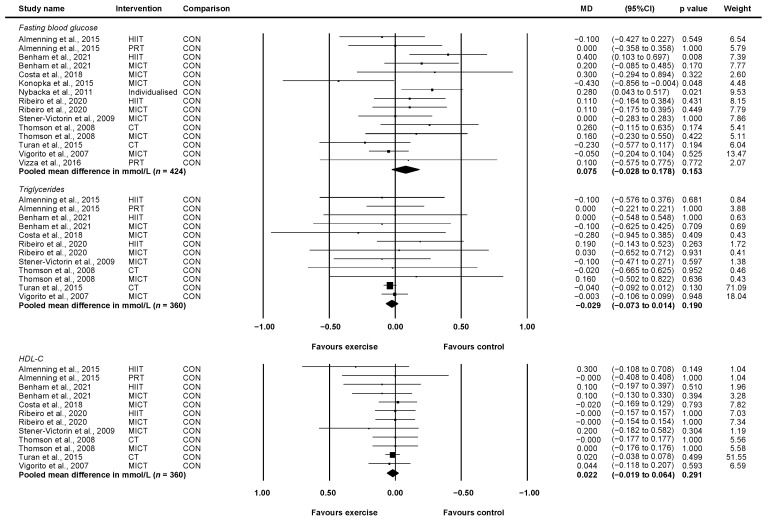
Effect of exercise vs. control for change in fasting blood glucose, HDL-C, and triglycerides. CI: confidence interval, CT: concurrent training, HDL-C: high-density lipoprotein cholesterol, HIIT: high-intensity interval training, MD: mean difference, MICT: moderate-intensity continuous training, PRT: progressive resistance training.

Eleven studies involving 424 unique participants reported fasting blood glucose in mmol/L or mg/dL [23,24,25,26,28,29,30,31,34,36,37]. Compared with control, exercise did not improve fasting blood glucose (WMD = 0.08 mmol/L, 95% CI: −0.03 to 0.18, *p* = 0.153, *I*^2^ = 37.38) (Figure 6). Two studies indicated potential publication bias following Egger’s test [26,31]. One of these studies involved a three-arm design for which only the HIIT group versus control indicated publication bias [26]. When these were removed in sensitivity analysis, the results remained unchanged despite a reduction in heterogeneity (WMD = 0.06 mmol/L, 95% CI: −0.02 to 0.14, *p* = 0.141, *I*^2^ = 2.38).

#### 3.4.6. Triglycerides

Eight studies involving 360 unique participants reported blood triglycerides in mmol/L or mg/dL [23,25,26,28,29,30,36,37]. Compared with control, exercise did not improve triglycerides (WMD = −0.03 mmol/L, 95% CI: −0.07 to 0.01, *p* = 0.190, *I*^2^ = 0) (Figure 6). There was no publication bias detected following Egger’s test. After removing two studies which weighed 71.09 and 18.04 [23,37], respectively, the results remained unchanged (WMD = 0.00 mmol/L, 95% CI: −0.13 to 0.13, *p* = 0.986, *I*^2^ = 0).

#### 3.4.7. HDL-C

Eight studies involving 360 unique participants reported HDL-C in mmol/L or mg/dL [23,25,26,28,29,30,36,37]. Compared with control, exercise did not improve HDL-C (WMD = 0.02 mmol/L, 95% CI: −0.02 to 0.06, *p* = 0.291, *I*^2^ = 0) (Figure 6). There was no publication bias detected following Egger’s test. After removing one study which weighed 51.55 [23], the results were unchanged (WMD = 0.02 mmol/L, 95% CI: −0.04 to 0.09, *p* = 0.413, *I*^2^ = 0). 

### 3.5. Sub-Analyses

A total of four studies reported sufficient data to determine the effect of HIIT vs. control [25,26,32,33] and five studies reported sufficient data to determine the effect of MICT vs. control [20,26,28,37,38] for change in cardiorespiratory fitness. Both HIIT and MICT significantly improved cardiorespiratory fitness when compared to control (HIIT: WMD = 2.87 mL/kg/min, 95% CI: 1.91 to 3.83, *p* < 0.001, *I*^2^ = 0, *n* = 113; MICT: WMD = 5.33 mL/kg/min, 95% CI: 4.08 to 6.58, *p* < 0.001, *I*^2^ = 15.84, *n* = 223). 

Five studies reported sufficient data to determine the effect of HIIT vs. control [25,26,29,33,35] and six studies reported sufficient data to determine the effect of MICT vs. control [21,26,28,29,30,37] for change in waist circumference. While HIIT led to greater pooled mean reductions in waist circumference than MICT, only MICT showed statistically significant improvements in waist reduction when compared to control (HIIT: WMD = −2.41 cm, 95% CI: −6.87 to 2.05, *p* = 0.290, *I*^2^ = 0, *n* = 157; MICT: WMD = −1.69 cm, 95% CI: −3.19 to −0.19, *p* = 0.027, *I*^2^ = 31.40, *n* = 278). 

Four studies reported sufficient data to determine the effect of HIIT vs. control [25,26,29,32] and five studies reported sufficient data to determine the effect of MICT vs. control [26,29,30,31,36] for change in HOMA-IR. Neither HIIT nor MICT improved HOMA-IR when compared to control (HIIT: WMD = −0.26, 95% CI: −0.77 to 0.25, *p* = 0.32, *I*^2^ = 0, *n* = 132; MICT: WMD = −0.20, 95% CI: −0.62 to 0.22, *p* = 0.35, *I*^2^ = 0, *n* = 153). 

Three studies reported sufficient data to determine the effect of HIIT vs. control [25,26,29] and seven studies reported sufficient data to determine the effect of MICT vs. control [26,28,29,30,31,36,37] for change in fasting blood glucose. Neither HIIT nor MICT improved fasting blood glucose when compared to control (HIIT: WMD = 0.14 mmol/L, 95% CI: −0.13 to 0.42, *p* = 0.31, *I*^2^ = 60.30, *n* = 102; MICT: WMD = 0.03, 95% CI: −0.11 to 0.16, *p* = 0.702, *I*^2^ = 28.02, *n* = 270). 

Three studies reported sufficient data to determine the effect of HIIT vs. control [25,26,29] and seven studies reported sufficient data to determine the effect of MICT vs. control [26,28,29,30,36,37] for change in triglycerides. Neither HIIT nor MICT improved triglycerides when compared to control (HIIT: WMD = 0.08 mmol/L, 95% CI; −0.17 to 0.32, *p* = 0.542, *I*^2^ = 0, *n* = 102; MICT: WMD = −0.01, 95% CI: −0.11 to 0.08, *p* = 0.765, *I*^2^ = 0, *n* = 245). 

Three studies reported sufficient data to determine the effect of HIIT vs. control [25,26,29] and seven studies reported sufficient data to determine the effect of MICT vs. control [26,28,29,30,36,37] for change in HDL-C. Neither HIIT nor MICT improved HDL-C when compared to control (HIIT: WMD = 0.05 mmol/L, 95% CI; −0.08 to 0.18, *p* = 0.449, *I*^2^ = 0, *n* = 102; MICT: WMD = 0.02, 95% CI: −0.05 to 0.10, *p* = 0.558, *I*^2^ = 0, *n* = 245). 

## 4. Discussion

The results from this systematic review and meta-analysis provide novel and up-to-date data regarding the effect of exercise interventions on key cardiometabolic risk factors in women with PCOS. Eighteen studies involving a total of 593 participants were combined in the meta-analyses, which showed that regular exercise significantly improved cardiorespiratory fitness and central obesity. The results also showed that exercise did not improve other cardiometabolic outcomes including systolic blood pressure, insulin resistance, blood glucose levels, and lipid profiles, albeit the baseline values were all within normal ranges. The results of the sub-analyses suggest that both HIIT and MICT interventions significantly improve cardiorespiratory fitness, and while both interventions also reduced waist circumference, only MICT led to statistically significant benefit.

Low cardiorespiratory fitness is a strong predictor of cardiometabolic risk [39] and all-cause mortality [40]. The results of this study showed that exercise significantly improved cardiorespiratory fitness by more than one metabolic equivalent (MET), equal to 3.5 mL/kg/min, when compared to a non-exercise control. These results indicate that exercise may play a critical part in cardiovascular health management for women with PCOS, who experience increased cardiovascular risk [7], as previous studies have shown that improvements in cardiorespiratory fitness of one MET were associated with a 13% risk reduction in all-cause mortality and a 15% reduction in the incidence of cardiovascular disease [41]. This finding is in accordance with an earlier meta-analysis which reported similar improvements in cardiorespiratory fitness of +3.84 mL/kg/min [42]. Furthermore, the results of the sub-analysis expand on previous findings by suggesting that both HIIT and MICT improve cardiorespiratory fitness, with MICT achieving greater improvements (HIIT: WMD = 2.87 mL/kg/min, *p* < 0.001; MICT: WMD = 5.33 mL/kg/min, *p* < 0.001). 

Waist circumference is included in the diagnostic criteria for the metabolic syndrome [43] and more accurately predicts cardiovascular risk than BMI [44], with a higher value also associated with an increased risk of developing type 2 diabetes and cardiovascular disease [45]. In fact, it is estimated that for every 1 cm increase in waist circumference, the relative risk of a cardiovascular event is estimated to increases by 2% [46]. The results of this study showed that exercise decreased waist circumference by −1.48 cm. Taken together, these results highlight the potential benefit of exercise for reducing the risk of cardiovascular disease-related events by approximately 3% through the reduction of waist circumference alone in women with PCOS. Furthermore, the results of the sub-analyses revealed that while both HIIT and MICT improved waist circumference, only MICT achieved statistical significance (HIIT: WMD = −2.41 cm, *p* = 2.05; MICT: WMD = −1.69 cm, *p* = 0.027). Importantly, while the magnitude of effect seen with exercise may not be large, the results support the utility of exercise as a therapeutic option for the management of central obesity, i.e., preventing central adiposity.

Previous reports have shown that young women with PCOS often present with elevated blood pressure independent of BMI [47]. In clinical practice, maintaining systolic blood pressure below 130 mmHg is the primary goal for individuals with, or at risk of, hypertension. To this end, lifestyle modification involving diet modulation and increased physical activity is the first line of therapy [48]. The results of this study show that exercise was associated with only modest non-significant reductions in systolic blood pressure when compared with control. Although the findings were non-significant, mean baseline systolic blood pressure across studies was 116 mmHg, which is considered normotensive (i.e., below 120 mmHg) [47]. 

Insulin resistance, which contributes to elevated fasting blood glucose levels, is highly prevalent in women with PCOS and significantly elevates the risk for ensuing type 2 diabetes [49]. Yet, fasting blood glucose, and not insulin resistance measured by HOMA-IR, is included in the diagnostic criteria for the metabolic syndrome [43]. The results of this study support the results of existing systematic reviews, which demonstrate that exercise does not significantly improve insulin resistance or fasting blood glucose levels beyond control in women with PCOS [42]. Furthermore, the results of the sub-analysis add to previous findings by showing that neither HIIT nor MICT improve fasting blood glucose levels. Importantly, these results may be explained by the normal baseline fasting blood glucose levels of participants included in the meta-analysis which averaged 4.93 mmol/L and were <5.44 mmol/L across all studies. As a result of these relatively normal values, exercise is not expected to further improve fasting blood glucose levels. Similarly, although exercise did not significantly improve insulin resistance, 10 of the 12 groups included in the meta-analysis favoured exercise for the reduction of insulin resistance when measured as HOMA-IR. Again, this non-significant result may be explained by the HOMA-IR baseline values averaging 2.67 across studies, which is on the cusp of normal. Furthermore, existing evidence supports the utility of exercise for improving glucose metabolism in individuals with more severe metabolic abnormalities, such as those with type 2 diabetes [50], and as a result, exercise may help prevent the onset of insulin resistance in women with PCOS.

Dyslipidaemia, involving abnormal circulating blood lipid levels, is associated with an array of cardiometabolic conditions such as type 2 diabetes [51], non-alcoholic fatty liver disease [52], and PCOS [53]. While the analyses undertaken in this review did not show a significant effect of exercise on HDL-C or triglyceride levels, all studies involved women with normal values for both HDL-C and triglycerides at baseline. As it is known that regular exercise improves lipid metabolism [54], it is reasonable to expect that dyslipidaemia in PCOS may be ameliorated following increased physical activity, yet further research is required to clarify this.

Previous systematic reviews have shown the efficacy of exercise for improving an array of cardiometabolic outcomes [55,56]. The results of this study go beyond previous reviews by providing up-to-date evidence surrounding the effects of exercise on cardiometabolic health in women with PCOS. Furthermore, the sub-analyses assessing the efficacy of HIIT and MICT on the aforementioned outcomes may be used to provide clinicians with more evidence-based prescription options when designing and delivering tailored exercise interventions. Importantly, the vast majority of studies included in this review had significant bias (Figure 2), and as a result, the certainty of evidence for the results generated was low at best (Table 4). Further high-quality studies are required to strengthen these findings and elucidate the role of exercise in the management of PCOS.

### 4.1. Implications of the Research

Although PCOS is associated with increased cardiometabolic risk factors, these may become exacerbated by comorbid and age-related factors such as reduced cardiorespiratory fitness and obesity. The results of this study show that regular exercise is an effective therapy for the management of multiple cardiometabolic risk factors including low cardiorespiratory fitness and abdominal adiposity in women with PCOS. While the results did not show a significant effect of exercise on systolic blood pressure, insulin resistance, fasting blood glucose levels, or lipid profiles, this result may be partly due to baseline values being within a relatively normal range. As cardiorespiratory fitness has been shown to be inversely associated with a range of cardiometabolic risk factors such as HOMA-IR, waist circumference, systolic blood pressure, and fasting glucose levels in individuals with obesity and type 2 diabetes [57], the results of the analyses indicate that exercise may be an effective strategy for the management of cardiometabolic risk in individuals with more severe metabolic abnormalities. Consequently, regular exercise may be useful for ameliorating the deterioration of cardiometabolic health in women with PCOS. Furthermore, while both HIIT and MICT improved cardiorespiratory fitness, MICT resulted in an almost two-fold greater increase. As a result, women with PCOS who are aiming to improve their cardiorespiratory fitness may see greater improvements by undertaking MICT rather than HIIT. While not as effective, HIIT may significantly improve cardiorespiratory fitness also, and this strategy may be particularly useful for time-poor individuals. Therefore, on the basis of the findings reported herein, as well as current available evidence, exercise appears to be an effective first-line therapy for the management of cardiometabolic health in women with PCOS. 

### 4.2. Limitations

This review has limitations that should be considered when interpreting the results. First, the majority of studies demonstrated unclear or high risk of bias on multiple domains as per the ROB2 assessment. As a result, the certainty of evidence was very low or low across all outcomes. Second, as the majority of studies included in the analysis incorporated sample sizes as low as five participants per group, there is a high risk for type II errors. Third, although compliance was high when reported, the impact of unreported and possibly poor adherence rates on the analyses undertaken could not be determined. As supervised exercise demonstrates greater improvements in adherence and overall fitness [58], studies with unsupervised sessions may differ in their adherence and intensity due to reliance on participant-reported measures. Fourth, because the research question was limited to women with PCOS and most studies were conducted in Western countries, the results may not be generalisable to other population groups. Fifth, given that women with PCOS can exhibit multiple phenotypes [59], each with possible variances in cardiometabolic risk, the paucity of data precluded our ability to determine the effect of exercise on specific PCOS phenotypes. Sixth, non-English language articles and grey literature were excluded which may result in publication bias, as was evident for some outcomes assessed in this review. Finally, although our meta-analysis suggests that exercise may improve surrogate markers of cardiometabolic health such as cardiorespiratory fitness over the short- to medium-term, the observed effect was modest. As such, further studies are required to determine whether exercise-induced improvements in health outcomes in women with PCOS can be maintained in the long-term. Despite these limitations, our review included only RCTs—the gold-standard design for determining causality. We incorporated a comprehensive search strategy conforming to international reporting guidelines, and performed sensitivity analyses where possible to account for high heterogeneity and publication bias. In doing so, we were able to provide an in-depth and up-to-date synthesis of current evidence regarding the effects of exercise interventions on cardiometabolic risk in women with PCOS.

## 5. Conclusions

The results of this study show that regular exercise is an effective therapy for the management of multiple cardiometabolic risk factors including low cardiorespiratory fitness and abdominal adiposity in women with PCOS. While HIIT and MICT interventions both improve cardiorespiratory fitness, the efficacy of HIIT for the management of other cardiometabolic risk factors requires further investigation. Additionally, because the majority of participants in this review did not have impaired fasting blood glucose levels or insulin resistance, further studies involving women with PCOS and more severe metabolic abnormalities should be undertaken to determine the efficacy of exercise in this subset of women with PCOS. 

## Figures and Tables

**Table 1:** Participant characteristics.

Study	Groups	Subjects	Age (Years) Mean ± SD	BMI (kg/m^2^) Mean ± SD	Diagnostic Criteria Used	Other Characteristics
Almenning et al., 2015 [25]	HIIT	8	NR	26.1 ± 6.5	Rotterdam 2003 or confirmation via general practitioner	Inactive adults
	RT	8	NR	27.4 ± 6.9		
	CON	9	NR	26.5 ± 5.0		
Benham et al., 2021 [26]	HIIT	12	29.1 ± 3.1	31.4 ± 8.6	Rotterdam 2003	Inactive adults
	MICT	12	29.5 ± 4.6	31.3 ± 9.0		
	CON	15	29.1 ± 5.4	31.6 ± 8.2		
Brown et al., 2009 [22]	MICT	8	NR	NR	≤8 menses per year and clinical or biochemical evidence of hyperandrogenism	Inactive, pre-menopausal adults aged 18–50
	CON	12	NR	NR		
Bruner et al., 2006 [27]	MICT + RT	7	32.3 ± 2.6	36.2 ± 5.3	Rotterdam 2003	Inactive adults with moderate and central obesity
	CON	5	28.4 ± 6	37.1 ± 7.6		
Costa et al., 2018 [28]	MICT	14	27.6 ± 4.5	32 ± 4.2	Rotterdam 2003	Inactive adults aged 18–34 with a BMI of 28–39.9 kg/m^2^
	CON	13	24.4 ± 5.0	33.6 ± 5.1		
Jedel et al., 2011 [20]	MICT	30	30.2 ± 4.7	27.7 ± 6.44	Ultrasound-verified polycystic ovaries, together with either oligo/amenorrhea and/or clinical signs of hyperandrogenism	Adults aged 18–37 with no pharmacological treatment 12 weeks before intervention
	CON	15	30.1 ± 4.2	26.8 ± 5.56		
Konopka et al., 2015 [31]	MICT	12	35 ± 5	33 ± 5	Rotterdam 2003	Inactive adults with insulin resistance and a BMI of 28–40 kg/m^2^
	CON	13				
Lionett et al., 2021 [33]	LV-HIIT	13	30 ± 7	29.8 ± 6.5	Rotterdam 2003	Adults aged 18–45, undertaking <2 weekly moderate-to-vigorous intensity endurance exercise sessions
	HIIT	14				
	CON	15				
Nybacka et al., 2011 [34]	MICT	12	31.1 ± 4.7	38.8 ± 7.9	Rotterdam 2003	Adults between 18 to 40 with a BMI > 27 kg/m^2^
	CON	14	29.3 ± 5.9	34.7 ± 5.0		
Ribeiro et al., 2020 [29]	HIIT	29	29.0 ± 4.3	28.7 ± 4.8	Rotterdam 2003	Inactive adultsaged 18–39
	MICT	28	29.1 ± 5.3	28.4 ± 5.6		
	CON	30	28.5 ± 5.8	29.1 ± 5.2		
Roessler et al., 2013 [35]	HIIT	8	31.0 ± 8.5	32.3 ± 7.4	Rotterdam 2003	Adults with a BMI of 25–40 kg/m^2^
	CON	9	36.7 ± 8.4	36.0 ± 6.9		
Samadi et al., 2019 [32]	HIIT	15	29.25 ± 2.80	32.8 ± 4.49	Rotterdam 2003	Adults aged 20–35 with insulin resistance and a BMI ≥ 30kg/m^2^
	CON	15	26.0 ± 4.38	34.06 ± 4.45		
Stener-Victorin et al. 2009 [36]	MICT	5	30.4 ± 5.5	26.8 ± 4.8	Rotterdam 2003	Adults aged 18–37
	CON	6	31.0 ± 3.2	28.0 ± 6.2		
Stener-Victorin et al. 2012 [21]	MICT	30	NR	NR	Rotterdam 2003	Adults aged 18–37
	CON	15	NR	NR		
Thomson et al., 2008 [30]	MICT	18	29.3 ± 6.8	36.1 ± 4.8	Rotterdam 2003	Inactive adults aged 18–41 with a BMI of 25–55 kg/m^2^
	MICT + RT	20				
	CON	14				
Turan et al., 2015 [23]	MICT + RT	14	24.45 ± 10.8	21.8 ± 3.7	Rotterdam 2003	Inactive adults aged 17–34 with BMI < 25 kg/m^2^
	CON	16		21.9 ± 4.4		
Vigorito et al., 2007 [37]	MICT	45	21.7 ± 2.3	29.3 ± 2.9	Rotterdam 2003	Adults with overweight or obesity
	CON	45	21.9 ± 1.9	29.4 ± 3.5		
Vizza et al., 2016 [24]	RT	7	26 ± 7	41.3 ± 12.5	None used. Diagnosis confirmed via the participant’s physician.	Adults aged 18–42 not participating in RT at time of recruitment
	CON	6	29 ± 3	34.0 ± 9.4		
Wu et al., 2021 [38]	MICT	19	32.7 ± 3.2	23.8 ± 3	Rotterdam 2003	Adults aged 18–40, undertaking physical exercise <3 times per week
	CON	19	33.2 ± 2.9	24.1 ± 3.2		

BMI: body mass index, CON: non-exercising control, HIIT: high-intensity interval training, LV-HIIT: low-volume high-intensity interval training, MICT: moderate-intensity continuous training, NR: not reported, RT: resistance training, SD: standard deviation.

**Table 2:** Exercise intervention details.

Study	Groups	Mode	Frequency (Days)	Intensity	Session Duration (Minutes)	Intervention Duration (Weeks)	Additional Intervention
Almenning et al., 2015 [25]	HIIT	Treadmill or outdoor walking/running and/or cycling (self-selected)	2/7	WU: 10 min at 70% HR_max_ HIIT: 4 × 4 min at 90–95% HR_max_ and 3 min at 70% HR_max_ CD: 5 min at 70% HR_max_	38	10	Participants in all groups advised to maintain usual diets
	RT	8 dynamic strength drills	3/7	75% 1RM for 3 sets of 10 repetitions, with 1 min rest between sets	NR		
	CON	Advised to adhere to ≥150 min of weekly moderate-intensity exercise without any follow-up during the ten-week intervention period					
Benham et al., 2021 [26]	HIIT	Aerobic exercise equipment of choice (e.g., treadmill, cycle ergometer, etc.)	3/7	WU: 5 min HIIT: 10 × 30 s at 90% HRR and 90 s of low-intensity aerobic exercise CD: 5 min	30	26	
	MICT	Aerobic exercise equipment of choice (e.g., treadmill, cycle ergometer, etc.)	3/7	WU: 5 min MICT: 40 min at 50–60% HRR CD: 5 min	50		
	CON	Participants in CON instructed to maintain usual level of physical activity					
Brown et al., 2009 [22]	MICT	Aerobic exercise equipment of choice (e.g., treadmill, cycle ergometer, etc.)	Dependent on bodyweight and VO_2peak_	14 kcal/kg/week at 50% VO_2peak_	Dependent on bodyweight and VO_2peak_, capped at 60 min every 24 h	12	Participants in both groups advised to maintain usual diets
	CON	No intervention					
Bruner et al., 2006 [27]	MICT + RT	Treadmill walking or stationary cycling	3/7	WU: 10 min MICT: 30 min at 70–85% HR_max_ CD: 10 min	40	12	Participants in both groups were encouraged to attend 1 h weekly seminars regarding long-term nutritional strategies
		Biceps curl, lat pulldown, leg curl, leg extension, shoulder press, chest press, leg press, hip abduction, hip adduction, hip flexion, hip extension, back extension		2 → 3 sets of 10 → 15 repetitions, with weight increasing by 5% or 2.2 kg. Encouraged to participate in physical activity (i.e., walking) on non-supervised days, and given an activity log to document this	90		
	CON	No exercise intervention					
Costa et al., 2018 [28]	MICT	Walking and/or jogging	3/7	WU: 5 min 40 min at: Weeks 1–4: 60–70% HR_max_ Weeks 5–8: 70–75% HR_max_ Weeks 9–12: 75–80% HR_max_ Weeks 13–16: 80–85% HR_max_ CD: 5 min	50	16	Participants in both groups advised to maintain usual diets
	CON	No intervention					
Jedel et al., 2011 [20]	MICT	Self-selected aerobic exercise, e.g., brisk walking, cycling	≥ 3/7	Self-selected pace faster than normal walking with HR of >120 bpm	30–45	16	Participants in both groups were given information regarding the importance of physical activity and healthy diet
	CON	No exercise intervention					
Konopka et al., 2015 [31]	MICT	Stationary cycling	5/7	60 min at 65% VO_2peak_	60	12	Participants were provided a standardised diet (50% carbohydrate, 30% fat, and 20% protein) three days prior to and for the duration of the study
	CON	No intervention					
Lionett et al., 2021 [33]	LV-HIIT	Treadmill or outdoor walking/running	3/7	WU: 10 min HIIT: 10 × 1 min at a maximally sustainable intensity, interspersed with 1 min of passive recovery or low-intensity walking CD: 3 min	32	16	
	HIIT	Treadmill or outdoor walking/running	3/7	WU: 10 min HIIT: 4 × 4 min at 90–95% HR_max,_ separated by 3 min of active recovery at ∼70% of HRmax CD: 3 min	38		
	CON	Participants in CON instructed to maintain usual level of physical activity, and informed about current recommendations for physical activity in adults					
Nybacka et al., 2011 [34]	Varied	Designed to enhance both the type and the level of physical activity to a level conforming to each individual patient’s capacity, goals, and interest at the beginning of the intervention	NR	NR	NR	17	Participants in both groups were asked to reduce daily energy intake by −600 kcal and maintain practices in accordance with Swedish nutritional recommendations
	CON						
Ribeiro et al., 2020 [29]	HIIT	Treadmill	3/7	WU: 5 min at 50–60% HR_max_ HIIT: 6 → 10 bouts of 2 min at 70–90% HR_max_ then 3 min at 60–70% HR_max_, with HR target increasing every 2–4 weeks CD: 5 min at 50–60% HR_max_	Weeks 1–3: 30 Weeks 4–6: 35 Weeks 7–10: 40 Weeks 11–13: 45 Weeks 14–16: 50	16	Participants in all groups advised to maintain usual diets
	MICT	Treadmill	3/7	WU: 5 min at 50–60% HR_max_ MICT: 65–80% HR_max_, gradually increasing every 2–4 weeks CD: 5 min at 50–60% HR_max_			
	CON	Advised to maintain daily physical activity profile					
Roessler et al., 2013 [35]	HIIT	Cycling and walking/running	3/7	WU: 15 min at 70–75% HR_max._ HIIT: repeated intervals of 0.5–5 min at 80–100% HR_max_ interspersed with 0.5 to 3 min rest at 45–65% HR_max_. CD: 5 min.	45	8	
	CON	Physical activity counselling	1/7				
Samadi et al., 2019 [32]	HIIT	Aquatic	3/7	WU: 5 min jogging and stretching HIIT: 4 × 4 min bouts of 8 × 20 s at maximal intensity followed by 10 s of rest at 80–95% HR_max_. 1 min of jogging at 75% HR_max_ was performed between each 4 min bout. CD: 5 min stretching	30	12	Participants in both groups took 3 pills of metformin (1500 mg) daily from the beginning of the intervention, and were advised to maintain usual diets
	CON	No regular exercises were performed					
Stener-Victorin et al., 2009 [36]	MICT	Self-selected aerobic exercise, e.g., brisk walking, cycling	≥3/7	Self-selected pace faster than normal walking with HR of >120 bpm	30–45	16	Participants in both groups were given information regarding the importance of physical activity and healthy diet
	CON	No exercise intervention					
Stener-Victorin et al., 2012 [21]	MICT	Self-selected aerobic exercise, e.g., brisk walking, cycling	≥3/7	Self-selected pace faster than normal walking with HR of >120 bpm	30–45	16	Participants in both groups were given information regarding the importance of physical activity and healthy diet
	CON	No exercise intervention					
Thomson et al., 2008 [30]	MICT	Walking/jogging	5/7	60–65% HR_max_ progressing to 75–80% HR_max_ over 20 weeks	25–30 progressing to 45 over 20 weeks	20	Participants were prescribed a diet of 5000–6000 kJ/d, with 30% protein, 40% carbohydrate, and 30% fat (<8% saturated fat)
	MICT + RT	Walking/jogging	3/7	60–65% HR_max_ progressing to 75–80% HR_max_ over 20 weeks	25–30 progressing to 45 over 20 weeks		
		Bench press, lat pulldown, leg press, knee extension, and sit-ups	2/7 on non-consecutive days	Weeks 1–2: 3 x 12 repetitions at 50–60% 1RM Weeks 3–20: 3 x 12 repetitions at 65–75% 1RM	3 x 12 repetitions of each exercise		
	CON	Dietary intervention only					
Turan et al., 2015 [23]	MICT + RT	Stepping	3/7	WU: 5 min walking on a treadmill at a low pace + static stretching MICT: 5–7 min → 20 min of stepping on a 10 cm-20 cm step at 10–15/20 RPE or 65–70% HR_max._ CD: 5 min walking on a treadmill at a low pace	50–60	8	Participants in both groups were given general dietary and behavioural advice, and prescribed a diet of 50% carbohydrates, 25% protein, and 25% fat
		Resistance band exercises targeting the back, trunk, and lower-body muscles	3/7	1 × 15 repetitions at 5–6/10 RPE with 30–60 s of rest between each exercise.			
	CON	Dietary intervention only					
Vigorito et al., 2007 [37]	MICT	Stationary cycling	3/7	WU: 5 min MICT: 30 min at 60–70% VO_2max_ CD: 5 min	40	12	Participants in both groups were counselled to achieve a healthy balanced meal plan with a nutritional composition in which 50% of the calories were from carbohydrate, 25% from protein, and 25% from fat
	CON	No intervention					
Vizza et al., 2016 [24]	RT	Lat pulldown, leg curl, seated row, leg press, calf raise, chest press, split squat, shoulder press, biceps curl, triceps extension and abdominal curl	2/7 non-consecutively	WU: 5 min on bicycle ergometer or treadmill RT: Performed to neuromuscular fatigue i.e., 8–12 RM; absolute loads increased with strength gains CD: 5 min on bicycle ergometer or treadmill	Weeks 1–2: 2 sets of each exercise Weeks 3–12: 3 sets of each exercise except spilt squats and shoulder press	12	
		Home-based calisthenics: hip rotations, side leg raises, push-ups on knees, wall squats, oblique curls, core stabilisation exercises	2/7 on days without supervised RT	NR	3 × 10 repetitions of each exercise		
	CON	Advised to continue current lifestyle					
Wu et al., 2021 [38]	MICT	Stationary cycling	4/7	WU: 15 min MICT: 30 min at VO*_2AT_* CD: 15 min	60	12	Participants in both groups advised to maintain usual diets
	CON						

1RM: one repetition maximum, bpm: beats/min, CD: cool-down, CON: non-exercising control, HIIT: high-intensity interval training, HR_max_: maximum heart rate, LV-HIIT: low-volume high-intensity interval training, MICT: moderate- to vigorous physical activity, NR: not reported, RM: repetition maximum, RPE: rate of perceived exertion, RT: resistance training, VO_2max_: maximum oxygen uptake, VO_2peak_: peak rate of oxygen uptake, VO*_2AT_*: maximum oxygen uptake at anaerobic threshold, WU: warm-up.

**Table 3:** Results of modified Downs and Black for methodological quality assessment.

Study	1	2	3	4	5	6	7	8	9	10	11	12	13	14	15	16	17	18	19	20	21	22	23	24	25	26	27	28	29	/29
Almenning et al., 2015 [25]	1	1	1	1	1	1	1	0	1	1	1	1	0	0	0	0	1	1	0	1	1	1	1	1	0	1	1	1	1	22
Benham et al., 2021 [26]	1	1	1	1	1	1	1	0	1	0	1	1	1	0	1	1	1	1	1	1	1	1	1	1	1	1	1	1	0	25
Brown et al., 2009 [22]	0	0	0	0	0	1	1	0	1	0	1	1	1	0	0	1	1	1	1	1	1	1	1	1	0	0	1	1	0	17
Bruner et al., 2006 [27]	1	1	1	1	0	1	1	0	0	0	1	1	0	0	0	0	1	1	1	1	1	1	1	0	0	0	0	0	1	16
Costa et al., 2018 [28]	1	1	1	1	1	1	1	0	1	1	1	1	1	0	0	1	1	1	1	1	1	1	0	0	0	1	1	1	1	23
Jedel et al., 2010 [20]	1	1	1	1	0	1	1	0	1	0	1	1	1	0	0	1	1	1	1	1	1	1	1	0	1	0	1	0	0	20
Konopka et al., 2015 [31]	0	1	1	1	1	1	1	0	0	0	1	1	0	0	0	0	1	1	1	1	1	1	1	0	1	0	1	0	1	18
Lionett et al., 2021 [33]	1	1	1	1	1	1	1	0	1	1	1	1	1	0	0	1	1	1	0	1	1	1	1	0	1	0	1	0	1	22
Nybacka et al., 2011 [34]	1	1	1	0	1	1	1	0	1	1	1	1	1	0	0	1	1	1	0	1	1	1	1	0	1	0	1	0	0	20
Ribeiro et al., 2020 [29]	1	1	1	1	1	1	1	0	1	1	0	0	0	0	0	1	1	1	1	1	1	1	1	0	1	0	1	1	1	21
Roessler et al., 2013 [35]	1	1	1	1	1	1	1	0	1	1	1	1	0	0	0	1	1	1	1	1	1	1	1	0	1	0	1	1	0	22
Samadi et al., 2019 [32]	1	1	1	1	0	1	1	0	0	1	0	0	0	0	0	1	1	1	0	1	1	1	1	0	1	0	1	0	0	16
Stener-Victorin et al., 2009 [36]	1	1	1	1	1	1	1	0	1	1	1	1	1	0	1	0	1	1	1	1	1	1	1	0	1	0	1	0	0	22
Stener-Victorin et al., 2012 [21]	1	1	1	1	1	1	1	0	0	1	1	1	1	0	1	1	1	1	1	1	1	1	1	1	1	0	1	0	0	23
Thomson et al., 2008 [30]	1	1	1	1	1	1	1	0	1	1	1	1	1	0	0	1	1	1	0	1	1	1	1	1	1	0	1	0	0	22
Turan et al., 2015 [23]	1	1	1	1	1	1	1	0	1	0	0	0	0	0	0	1	1	1	0	1	1	1	1	1	1	1	1	1	1	21
Vigorito et al., 2007 [37]	1	1	1	1	1	1	1	0	0	1	0	0	0	0	1	1	1	1	0	1	1	1	1	0	1	1	1	1	1	21
Vizza et al., 2016 [24]	1	1	1	1	1	1	1	0	1	1	1	1	0	0	0	0	1	1	0	1	1	1	1	1	1	1	1	1	1	23
Wu et al., 2021 [38]	1	1	1	1	1	1	1	0	0	1	1	1	1	0	0	1	1	1	0	1	1	1	1	0	1	0	1	0	1	21

**Table 4:** Assessment of certainty of evidence summary.

Exercise compared to non-exercise control for women with PCOS					
Patient or population: women with PCOS Setting: Intervention: exercise Comparison: non-exercise control					
Outcomes	Anticipated absolute effects * (95% CI)		No. of participants (studies)	Certainty of evidence (GRADE)	Comments
	Score with control	Score with exercise			
Cardiorespiratory fitness (reported in ml/kg/min)	Mean VO_2max_ = 29.50 mL/kg/min	MD 4.00 mL/kg/min higher (2.61 higher to 5.40 higher)	343 (9)	⊕⊕◯◯ ^a^ Low	Exercise may increase cardiorespiratory fitness in women with PCOS.
Waist circumference (reported in cm)	Mean waist circumference = 95.93 cm	MD 1.48 cm lower (2.35 lower to 0.62 lower)	462 (12)	⊕⊕◯◯ ^b^ Low	Exercise may elicit modest reductions in waist circumference in women with PCOS.
Systolic blood pressure (reported in mmHg)	Mean blood pressure = 116.24 mmHg	MD 1.88 mmHg lower (5.09 lower to 1.34 higher)	282 (6)	⊕◯◯◯ ^c,d^ Very low	It is unlikely that exercise elicits meaningful changes in systolic blood pressure in women with PCOS (and normal blood pressure) but we are very uncertain.
HOMA-IR	Mean HOMA-IR index = 2.69	MD 0.17 lower (0.44 lower to 0.09 higher)	337 (10)	⊕⊕◯◯ ^e^ Low	It is unlikely that exercise elicits meaningful changes in HOMA-IR in women with PCOS.
Fasting blood glucose (reported in mmol/L)	Mean fasting blood glucose = 4.93 mmol/L	MD 0.08 mmol/L higher (0.03 lower to 0.18 higher)	424 (11)	⊕⊕◯◯ ^f^ Low	It is unlikely that exercise elicits meaningful changes in fasting blood glucose in women with PCOS (and normal blood glucose).
Triglycerides (reported in mmol/L)	Mean blood triglycerides = 1.24 mmol/L	MD 0.03 mmol/L lower (0.07 lower to 0.01 higher)	360 (8)	⊕⊕◯◯ ^g^ Low	It is unlikely that exercise elicits meaningful changes in blood triglycerides in women with PCOS (and normal blood triglyceride levels).
HDL-C (reported in mmol/L)	Mean HDL-C = 1.30 mmol/L	MD 0.02 mmol/L higher (0.02 lower to 0.06 higher)	360 (8)	⊕⊕◯◯ ^g^ Low	It is unlikely that exercise elicits meaningful changes in HDL-C in women with PCOS (and normal HDL-C).
* The score in the intervention group (and its 95% CI) is based on the assumed score in the comparison group. PCOS: Polycystic Ovary Syndrome, CI: confidence interval, GRADE: Grading of Recommendations, Assessment, Development, and Evaluation, VO_2max_: maximal oxygen uptake, MD: mean difference, HOMA-IR: homeostatic model assessment of insulin resistance, HDL-C: high-density lipoprotein cholesterol.					
**GRADE Working Group grades of evidence** *High certainty:* We are very confident that the true effect lies close to that of the estimate of the effect. *Moderate certainty:* We are moderately confident in the effect estimate: The true effect is likely to be close to the estimate of the effect, but there is a possibility that it is substantially different. *Low certainty:* Our confidence in the effect estimate is limited: The true effect may be substantially different from the estimate of the effect. *Very low certainty:* We have very little confidence in the effect estimate: The true effect is likely to be substantially different from the estimate of effect.					
**Explanations** ^a^ Downgraded two levels for serious risk of bias: 7 of 9 included studies had an unclear or high risk of bias for blinding of outcome assessment, and 5 of 9 included studies had an unclear or high risk of bias for allocation concealment and incomplete outcome data, respectively. ^b^ Downgraded three levels for serious risk of bias: 10 of 12 included studies had an unclear or high risk of bias for blinding of outcome assessment, 6 of 12 included studies had an unclear or high risk of bias for allocation concealment, 7 of 12 studies had an unclear or high risk of bias for selective reporting, and 6 of 12 included studies did not report intervention adherence. ^c^ Downgraded two levels for serious risk of bias: 4 of 6 included studies had an unclear or high risk of bias for blinding of outcome assessment, 4 of 6 included studies had an unclear or high risk of bias for allocation concealment, and 4 of 6 included studies had an unclear or high risk of bias for selective reporting. ^d^ Downgraded one level for serious imprecision: small sample size. ^e^ Downgraded two levels for serious risk of bias: 8 of 10 included studies had an unclear or high risk of bias for blinding of outcome assessment, 6 of 10 included studies had an unclear or high risk of bias for incomplete outcome data, and 5 of 10 studies did not report intervention adherence. ^f^ Downgraded two levels for serious risk of bias: 8 of 11 included studies had an unclear or high risk of bias for blinding of outcome assessment, 6 of 11 included studies had an unclear or high risk of bias for allocation concealment and selective reporting, respectively. ^g^ Downgraded two levels for serious risk of bias: 5 of 8 included studies had an unclear or high risk of bias for blinding of outcome assessment, 4 of 8 included studies had an unclear or high risk of bias for allocation concealment and selective reporting, respectively.					

## Data Availability

The datasets used and/or analysed during the current study are available from the corresponding author on reasonable request.

## Appendix A

Methodological quality assessment modified Downs and Black checklist.

Reporting	Score
1. Is the hypothesis/aim/objective of the study clearly described?	0–1
2. Are the main outcomes to be measured clearly described in the Introduction or Methods section?	0–1
3. Are the characteristics of the patients included in the study clearly described?	0–1
4. Are the interventions of interest clearly described?	0–1
5. Are the distributions of principal confounders in each group of subjects to be compared clearly described?	0–1
6. Are the main findings of the study clearly described?	0–1
7. Does the study provide estimates of the random variability in the data for the main outcomes?	0–1
8. Have all important adverse events that may be a consequence of the intervention been reported?	0–1
9. Have the characteristics of patients lost to follow-up been described?	0–1
10. Have actual probability values been reported (e.g., 0.035 rather than <0.05) for the main outcomes except where the probability value is less than 0.001?	0–1
External validity	
11. Were the subjects asked to participate in the study representative of the entire population from which they were recruited?	0–1
12. Were those subjects who were prepared to participate representative of the entire population from which they were recruited?	0–1
13. Were the staff, places, and facilities where the patients were treated representative of the treatment the majority of patients received?	0–1
Internal validity—bias	
14. Was an attempt made to blind study subjects to the intervention they have received?	0–1
15. Was an attempt made to blind those measuring the main outcomes of the intervention?	0–1
16. If any of the results of the study were based on “data dredging”, was this made clear?	0–1
17. In trials and cohort studies, do the analyses adjust for different lengths of follow-up of patients, or in case-control studies, is the time period between the intervention and outcome the same for cases and controls?	0–1
18. Were the statistical tests used to assess the main outcomes appropriate?	0–1
19. Was compliance with the intervention/s reliable?	0–1
20. Were the main outcome measures used accurate (valid and reliable)?	0–1
Internal validity—confounding (selection bias)	
21. Were the patients in different intervention groups (trials and cohort studies), or were the cases and controls (case-control studies) recruited from the same population?	0–1
22. Were study subjects in different intervention groups (trials and cohort studies), or were the cases and controls (case-control studies) recruited over the same period of time?	0–1
23. Were study subjects randomised to intervention groups?	0–1
24. Was the randomised intervention assignment concealed from both patients and health care staff until recruitment was complete and irrevocable?	0–1
25. Was there adequate adjustment for confounding in the analyses from which the main findings were drawn?	0–1
26. Were losses of patients to follow-up taken into account?	0–1
Study power	
27. Did the study have sufficient power to detect a clinically important effect where the probability value for a difference being due to change is less than 5%?	0–1
Exercise adherence and supervision	
28. Was exercise adherence reported?	0–1
29. Were exercise sessions supervised?	0–1

## References

[1] Deswal R., Narwal V., Dang A., Pundir C.S. (2020). The Prevalence of Polycystic Ovary Syndrome: A Brief Systematic Review. J. Hum. Reprod. Sci..

[2] Teede H.J., Hutchison S., Zoungas S., Meyer C. (2006). Insulin resistance, the metabolic syndrome, diabetes, and cardiovascular disease risk in women with PCOS. Endocrine.

[3] Lim S.S., Davies M.J., Norman R.J., Moran L.J. (2012). Overweight, obesity and central obesity in women with polycystic ovary syndrome: A systematic review and meta-analysis. Hum. Reprod. Updat..

[4] DeUgarte C.M., Bartolucci A.A., Azziz R. (2005). Prevalence of insulin resistance in the polycystic ovary syndrome using the homeostasis model assessment. Fertil. Steril..

[5] Cassar S., Misso M.L., Hopkins W.G., Shaw C.S., Teede H.J., Stepto N.K. (2016). Insulin resistance in polycystic ovary syndrome: A systematic review and meta-analysis of euglycaemic-hyperinsulinaemic clamp studies. Hum. Reprod..

[6] Gambineri A., Patton L., Altieri P., Pagotto U., Pizzi C., Manzoli L., Pasquali R. (2012). Polycystic ovary syndrome is a risk factor for type 2 diabetes: Results from a long-term prospective study. Diabetes.

[7] Osibogun O., Ogunmoroti O., Michos E.D. (2020). Polycystic ovary syndrome and cardiometabolic risk: Opportunities for cardiovascular disease prevention. Trends Cardiovasc. Med..

[8] Donà S., Bacchi E., Moghetti P. (2017). Is cardiorespiratory fitness impaired in PCOS women? A review of the literature. J. Endocrinol. Investig..

[9] Teede H.J., Misso M.L., Costello M.F., Dokras A., Laven J., Moran L., Piltonen T., Norman R.J., on behalf of theInternational PCOS Network (2018). Recommendations from the international evidence-based guideline for the assessment and management of polycystic ovary syndrome. Fertil. Steril..

[10] Sabag A., Little J.P., Johnson N.A. (2021). Low-volume high-intensity interval training for cardiometabolic health. J. Physiol..

[11] Liberati A., Altman D.G., Tetzlaff J., Mulrow C., Gøtzsche P.C., Ioannidis J.P.A., Clarke M., Devereaux P.J., Kleijnen J., Moher D. (2009). The PRISMA statement for reporting systematic reviews and meta-analyses of studies that evaluate healthcare interventions: Explanation and elaboration. BMJ.

[12] Eshre T.R., Group A-SPCW (2004). Revised 2003 consensus on diagnostic criteria and long-term health risks related to polycystic ovary syndrome. Fertil. Steril..

[13] Zawadzski J. (1992). Diagnostic criteria for polycystic ovary syndrome: Towards a rational approach. Polycystic Ovary Syndr..

[14] Azziz R., Carmina E., Dewailly D., Diamanti-Kandarakis E., Escobar-Morreale H., Futterweit W., Janssen O.E., Legro R., Norman R., Taylor A.E. (2006). Criteria for defining polycystic ovary syndrome as a predominantly hyperandrogenic syndrome: An androgen excess society guideline. J. Clin. Endocrinol. Metab..

[15] Norton K., Norton L., Sadgrove D. (2010). Position statement on physical activity and exercise intensity terminology. J. Sci. Med. Sport.

[16] Higgins J.P., Thompson S.G., Deeks J.J., Altman D.G. (2003). Measuring inconsistency in meta-analyses. BMJ.

[17] Downs S.H., Black N. (1998). The feasibility of creating a checklist for the assessment of the methodological quality both of randomised and non-randomised studies of health care interventions. J Epidemiol. Community Health.

[18] Sterne J.A.C., Savović J., Page M.J., Elbers R.G., Blencowe N.S., Boutron I., Cates C.J., Cheng H.Y., Corbett M.S., Eldridge S.M. (2019). RoB 2: A revised tool for assessing risk of bias in randomised trials. BMJ.

[19] Guyatt G.H., Oxman A.D., Schünemann H.J., Tugwell P., Knottnerus A. (2011). GRADE guidelines: A new series of articles in the Journal of Clinical Epidemiology. J. Clin. Epidemiol..

[20] Jedel E., Labrie F., Odén A., Holm G., Nilsson L., Janson P.O., Lind A.-K., Ohlsson C., Stener-Victorin E. (2011). Impact of electro-acupuncture and physical exercise on hyperandrogenism and oligo/amenorrhea in women with polycystic ovary syndrome: A randomized controlled trial. Am. J. Physiol. Endocrinol. Metab..

[21] Stener-Victorin E., Baghaei F., Holm G., Janson P.O., Olivecrona G., Lönn M., Mannerås-Holm L. (2012). Effects of acupuncture and exercise on insulin sensitivity, adipose tissue characteristics, and markers of coagulation and fibrinolysis in women with polycystic ovary syndrome: Secondary analyses of a randomized controlled trial. Fertil. Steril..

[22] Brown A.J., Setji T.L., Sanders L.L., Lowry K.P., Otvos J.D., E Kraus W., Svetkey P.L. (2009). Effects of exercise on lipoprotein particles in women with polycystic ovary syndrome. Med. Sci. Sports Exerc..

[23] Turan V., Mutlu E.K., Solmaz U., Ekin A., Tosun O., Tosun G., Mat E., Gezer C., Malkoç M. (2015). Benefits of short-term structured exercise in non-overweight women with polycystic ovary syndrome: A prospective randomized controlled study. J. Phys. Ther. Sci..

[24] Vizza L., Smith C.A., Swaraj S., Agho K., Cheema B.S. (2016). The feasibility of progressive resistance training in women with polycystic ovary syndrome: A pilot randomized controlled trial. BMC Sports Sci. Med. Rehabil..

[25] Almenning I., Rieber-Mohn A., Lundgren K.M., Shetelig Løvvik T., Garnæs K.K., Moholdt T. (2015). Effects of High Intensity Interval Training and Strength Training on Metabolic, Cardiovascular and Hormonal Outcomes in Women with Polycystic Ovary Syndrome: A Pilot Study. PLoS ONE.

[26] Benham J.L., Booth J.E., Corenblum B., Doucette S., Friedenreich C.M., Rabi D.M., Sigal R.J. (2021). Exercise training and reproductive outcomes in women with polycystic ovary syndrome: A pilot randomized controlled trial. Clin. Endocrinol. (Oxf)..

[27] Bruner B., Chad K., Chizen D. (2006). Effects of exercise and nutritional counseling in women with polycystic ovary syndrome. Appl Physiol. Nutr. Metab..

[28] Costa E.C., DE Sá J.C.F., Stepto N.K., Costa I.B.B., Farias Junior L.F., Moreira S.D.N.T., Soares E.M.M., Lemos T.M.A.M., Browne R.A.V., De Azevedo G.D. (2018). Aerobic Training Improves Quality of Life in Women with Polycystic Ovary Syndrome. Med. Sci. Sports Exerc..

[29] Ribeiro V.B., Kogure G.S., Lopes I.P., Silva R.C., Pedroso D.C.C., De Melo A.S., de Souza H.C.D., Ferriani R.A., Miranda Furtado C.L., Dos Reis R.M. (2020). Effects of continuous and intermittent aerobic physical training on hormonal and metabolic profile, and body composition in women with polycystic ovary syndrome: A randomized controlled trial. Clin. Endocrinol. (Oxf)..

[30] Thomson R.L., Buckley J.D., Noakes M., Clifton P.M., Norman R.J., Brinkworth G.D. (2008). The effect of a hypocaloric diet with and without exercise training on body composition, cardiometabolic risk profile, and reproductive function in overweight and obese women with polycystic ovary syndrome. J. Clin. Endocrinol. Metab..

[31] Konopka A.R., Asante A., Lanza I.R., Robinson M.M., Johnson M.L., Man C.D., Cobelli C., Amols M.H., Irving B.A., Nair K. (2015). Defects in mitochondrial efficiency and H2O2 emissions in obese women are restored to a lean phenotype with aerobic exercise training. Diabetes.

[32] Samadi Z., Bambaeichi E., Valiani M., Shahshahan Z. (2019). Evaluation of Changes in Levels of Hyperandrogenism, Hirsutism and Menstrual Regulation After a Period of Aquatic High Intensity Interval Training in Women with Polycystic Ovary Syndrome. Int. J. Prev. Med..

[33] Lionett S., Kiel I.A., Røsbjørgen R., Lydersen S., Larsen S., Moholdt T. (2021). Absent Exercise-Induced Improvements in Fat Oxidation in Women With Polycystic Ovary Syndrome After High-Intensity Interval Training. Front. Physiol..

[34] Nybacka Å., Carlström K., Ståhle A., Nyrén S., Hellström P.M., Hirschberg A.L. (2011). Randomized comparison of the influence of dietary management and/or physical exercise on ovarian function and metabolic parameters in overweight women with polycystic ovary syndrome. Fertil. Steril..

[35] Roessler K.K., Birkebaek C., Ravn P., Andersen M.S., Glintborg D. (2013). Effects of exercise and group counselling on body composition and VO2max in overweight women with polycystic ovary syndrome. Acta Obstet. Gynecol. Scand..

[36] Stener-Victorin E., Jedel E., Janson P.O., Sverrisdottir Y.B. (2009). Low-frequency electroacupuncture and physical exercise decrease high muscle sympathetic nerve activity in polycystic ovary syndrome. Am. J. Physiol. Regul. Integr. Comp. Physiol..

[37] Vigorito C., Giallauria F., Palomba S., Cascella T., Manguso F., Lucci R., De Lorenzo A., Tafuri D., Lombardi G., Colao A. (2007). Beneficial effects of a three-month structured exercise training program on cardiopulmonary functional capacity in young women with polycystic ovary syndrome. J. Clin. Endocrinol. Metab..

[38] Wu X., Wu H., Sun W., Wang C. (2021). Improvement of anti-Müllerian hormone and oxidative stress through regular exercise in Chinese women with polycystic ovary syndrome. Hormones (Athens).

[39] Knaeps S., Lefevre J., Wijtzes A., Charlier R., Mertens E., Bourgois J.G. (2016). Independent Associations between Sedentary Time, Moderate-To-Vigorous Physical Activity, Cardiorespiratory Fitness and Cardio-Metabolic Health: A Cross-Sectional Study. PLoS ONE.

[40] Lyerly G.W., Sui X., Lavie C.J., Church T.S., Hand G.A., Blair S.N. (2009). The association between cardiorespiratory fitness and risk of all-cause mortality among women with impaired fasting glucose or undiagnosed diabetes mellitus. Mayo. Clin. Proc..

[41] Kodama S., Saito K., Tanaka S., Maki M., Yachi Y., Asumi M., Sugawara A., Totsuka K., Shimano H., Ohashi Y. (2009). Cardiorespiratory fitness as a quantitative predictor of all-cause mortality and cardiovascular events in healthy men and women: A meta-analysis. JAMA.

[42] Kite C., Lahart I.M., Afzal I., Broom D.R., Randeva H., Kyrou I., Brown J.E. (2019). Exercise, or exercise and diet for the management of polycystic ovary syndrome: A systematic review and meta-analysis. Syst. Rev..

[43] Heart L.N., Grundy S.M., Cleeman J.I., Daniels S.R., Donato K.A., Eckel R.H., A Franklin B., Gordon D.J., Krauss R.M., American Heart Association (2005). Diagnosis and management of the metabolic syndrome: An American Heart Association/National Heart, Lung, and Blood Institute Scientific Statement. Circulation.

[44] E Staiano A., A Reeder B., Elliott S., Joffres M.R., Pahwa P., A Kirkland S., Paradis G., Katzmarzyk P.T. (2012). Body mass index versus waist circumference as predictors of mortality in Canadian adults. Int. J. Obes..

[45] Janiszewski P.M., Janssen I., Ross R. (2007). Does waist circumference predict diabetes and cardiovascular disease beyond commonly evaluated cardiometabolic risk factors?. Diabetes Care.

[46] De Koning L., Merchant A.T., Pogue J., Anand S.S. (2007). Waist circumference and waist-to-hip ratio as predictors of cardiovascular events: Meta-regression analysis of prospective studies. Eur. Heart J..

[47] Joham A.E., Boyle J.A., Zoungas S., Teede H.J. (2015). Hypertension in Reproductive-Aged Women With Polycystic Ovary Syndrome and Association With Obesity. Am. J. Hypertens..

[48] Jones D.W., Whelton P.K., Allen N., Clark D., Gidding S.S., Muntner P., Nesbitt S., Mitchell N.S., Townsend R., Falkner B. (2021). Management of Stage 1 Hypertension in Adults With a Low 10-Year Risk for Cardiovascular Disease: Filling a Guidance Gap: A Scientific Statement From the American Heart Association. Hypertension.

[49] Boudreaux M.Y., Talbott E.O., Kip K.E., Brooks M.M., Witchel S.F. (2006). Risk of T2DM and impaired fasting glucose among PCOS subjects: Results of an 8-year follow-up. Curr. Diab. Rep..

[50] Umpierre D., Ribeiro P.A., Kramer C.K., Leitão C.B., Zucatti A.T., Azevedo M.J., Gross J.L., Ribeiro J.P., Schaan B.D. (2011). Physical activity advice only or structured exercise training and association with HbA1c levels in type 2 diabetes: A systematic review and meta-analysis. JAMA.

[51] Schofield J.D., Liu Y., Rao-Balakrishna P., Malik R.A., Soran H. (2016). Diabetes Dyslipidemia. Diabetes Ther..

[52] Chen Z., Qin H., Qiu S., Chen G., Chen Y. (2019). Correlation of triglyceride to high-density lipoprotein cholesterol ratio with nonalcoholic fatty liver disease among the non-obese Chinese population with normal blood lipid levels: A retrospective cohort research. Lipids Health Dis..

[53] Kim J.J., Choi Y.M. (2013). Dyslipidemia in women with polycystic ovary syndrome. Obstet. Gynecol. Sci..

[54] Mika A., Macaluso F., Barone R., Di Felice V., Sledzinski T. (2019). Effect of Exercise on Fatty Acid Metabolism and Adipokine Secretion in Adipose Tissue. Front. Physiol..

[55] Benham J.L., Yamamoto J.M., Friedenreich C.M., Rabi D.M., Sigal R.J. (2018). Role of exercise training in polycystic ovary syndrome: A systematic review and meta-analysis. Clin. Obes..

[56] Dos Santos I.K., Ashe M.C., Cobucci R.N., Soares G.M., De Oliveira Maranhão T.M., Dantas P.M.S. (2020). The effect of exercise as an intervention for women with polycystic ovary syndrome: A systematic review and meta-analysis. Medicine (Baltim.).

[57] Sabag A., Keating S.E., Way K.L., Sultana R.N., Lanting S.M., Twigg S.M., Johnson N.A. (2021). The association between cardiorespiratory fitness, liver fat and insulin resistance in adults with or without type 2 diabetes: A cross-sectional analysis. BMC Sports Sci. Med. Rehabil..

[58] Fennell C., Peroutky K., Glickman E. (2016). Effects of supervised training compared to unsupervised training on physical activity, muscular endurance, and cardiovascular parameters. MOJ Orthop. Rheumatol..

[59] Bozdag G., Mumusoglu S., Zengin D., Karabulut E., Yildiz B.O. (2016). The prevalence and phenotypic features of polycystic ovary syndrome: A systematic review and meta-analysis. Hum. Reprod..

